# On safety, pharmacokinetics and dosage of bevacizumab in ROP treatment – a review

**DOI:** 10.1111/j.1651-2227.2011.02445.x

**Published:** 2011-12

**Authors:** Anna-Lena Hård, Ann Hellström

**Affiliations:** Department of Ophthalmology, Institute of Neuroscience and Physiology, The Sahlgrenska Academy at University of GothenburgGothenburg, Sweden

**Keywords:** Anti-vascular endothelial growth factor, Bevacizumab, Pharmacokinetics, Retinopathy of prematurity

## Abstract

Off-label intravitreal use of the vascular endothelial growth factor (VEGF) antibody bevacizumab for retinopathy of prematurity (ROP) increases despite lack of studies on safety, pharmacokinetics and dosage in developing individuals. Systemic absorption has been considered negligible. A literature search was performed with emphasis on potential adverse systemic effects in developing individuals.

**Conclusion:**

Intravitreal bevacizumab enters the general circulation, suppresses plasma VEGF levels and remains in the blood for more than 8 weeks in primates. Possible adverse effects on VEGF-dependent development must be considered.


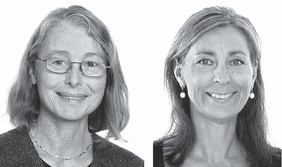


## Introduction

The number of publications on the off-label use of intravitreal bevacizumab (Avastin®, Roche, Basel, Switzerland) for severe retinopathy of prematurity (ROP) is rapidly increasing. Although most authors agree that studies of pharmacokinetics and systemic safety are needed, no such studies on preterm infants have been published. A recent editorial expressed the opinion that it seems reasonable to assume that intravitreal bevacizumab is safe and that it should be the treatment of choice for zone I ROP (1). Others question the use of this medication without clinical trials with meticulous evaluation of multiple variables that normally precedes the introduction of drugs in clinical use (2,3).

One randomized controlled study of intravitreal use of bevacizumab (BEAT-ROP) has been published so far (4). The dose injected was 0.625 mg, i.e., half the dose used in adults. Regarding safety, it was concluded that for assessment of mortality, 2800 infants would be required and assessment of local or systemic toxicity would require an even larger number of infants. It was stated that bevacizumab could not escape the eye more than in very small amounts because of its large size, unless laser therapy had destroyed the retinal barrier.

**Key notes**

- Intravitreal bevacizumab enters the general circulation, results in prolonged VEGF inhibition and has a half-life of 1–2 weeks in primates. VEGF is critical for growth and development of vital organs such as kidneys, lungs and brain during the third trimester. After proper investigations of systemic effects, pharmacokinetics and dosage, anti-VEGF might be an opportunity for severe ROP. As an alternative to laser, its effects are presently too poorly known.

A prematurely born infant receiving treatment for ROP is at a stage when growth and differentiation normally are intense. Early development is characterized by critical periods of susceptibility when environmental factors effectively produce long-lasting changes. Knowing that vascular endothelial growth factor (VEGF) is essential for normal angiogenesis and, in addition, has neuroprotective effects, we set out to review studies on safety, pharmacokinetics and dosage of the drug in relation to the developmental stages of important organs during the third trimester and early postnatal life.

Bevacizumab is a recombinant humanized vascular endothelial VEGF antibody that prevents VEGF from binding to its receptors (5). Bevacizumab binds to all isoforms of VEGF (6), blocks VEGF-induced angiogenesis and is approved by the U.S. Food and Drug administration for intravenous use for metastatic colorectal cancer. It is used off-label intravitreal to treat neovascular retinal disorders such as age-related macular degeneration (7), diabetic retinopathy (8) and central retinal vein occlusion (9).

Metabolism and elimination of bevacizumab are similar to those of endogeneous IgG, i.e., primarily via proteolytic catabolism in the whole body including endothelial cells and not mainly through the kidneys or the liver (FASS.se).

Vascular endothelial growth factor, a secreted glycoprotein, is an angiogenic as well as a vasopermeable factor which is secreted by foetal and adult epithelial and mesenchymal cells and exerts mitogenic effects on endothelial cells. In the foetus, VEGF is expressed in most tissues. In normal angiogenesis, VEGF activity often represents a rate-limiting step. Median plasma concentrations of VEGF in premature babies in one study showed a large variation but no significant difference between infants without and with ROP at 32 weeks postmenstrual age (PMA) (median 0. 658 ng/mL, range 0.049–2.152 and median 0.904, range 0.142–2.349, respectively) and at 36 weeks PMA (median 0.437, range 0.089–2.367 and 0.344, range 0.066–1.334 ng/mL, respectively) (10). In the human *kidney*, VEGF is highly expressed during glomerular development and also in the adult indicating roles for normal glomerulogenesis and for control of vascular permeability (11). A strong dosage sensitivity for VEGF-A in the developing glomerulus has been reported, and dysregulation of VEGF has been found to play a pathogenic role in glomerular disease. A note of caution for clinical trials aimed at altering VEGF levels has been issued and careful monitoring of renal function with a particular emphasis on the glomerular filtration barrier is recommended (12).

In the human *lung*, primitive alveoli are first seen at PMA 29 weeks. With increasing gestation, the alveoli get thinner walls, and at 36 weeks, all alveoli are thin walled (13). There is strong evidence that VEGF is necessary for alveolarization during normal lung development and that inhibition of VEGF during a critical period of growth contributes to bronchopulmonary dysplasia (BPD) (14). In a study of premature mice, VEGF increased surfactant synthesis and improved lung function and was considered a potential therapeutic possibility for respiratory distress syndrome (15).

In a study on human foetal and postnatal *brains*, VEGF expression was found in different locations during different time periods. Bevacizumab treatment for ROP mainly takes place at PMA 30–40 weeks. At that time, VEGF expression was found in some brain locations but not in others (16).

## Dosage, pharmacokinetics and safety

### In vitro

In the following, all concentrations of VEGF and bevacizumab in blood will be expressed as ng/mL for simplicity.

Wang et al. (17) studied bevacizumab-induced inhibition of VEGF (50 ng/mL)-mediated effects on human umbilical vein endothelial cells (HUVECs) in cultures. They found a dose-dependent inhibition of VEGF-induced HUVEC proliferation with an estimated half maximal inhibitory concentration (IC_50_) of 22 ng/mL. The addition of 500 ng/mL of bevacizumab completely blocked VEGF-induced endothelial cell growth, suggesting that a molar ratio of bevacizumab to VEGF of 2.6:1 is needed for maximum inhibition. Also for blockage of HUVEC survival, nitric oxide (NO) production and permeability, a ratio of 2.6:1 was efficient, while a ratio of 10:1 was needed to block migration.

Porchine VEGF binds to bevacizumab. In perfused organ cultures from pig's eyes, where 0.35 ng/mL of VEGF was produced per hour, VEGF was completely neutralized for 16 h by 0.25 mg/mL of bevacizumab and 0.125 mg/mL of ranibizumab. The efficiency of the two drugs was similar (18).

### Mice and rats

Mice and rat VEGF lack affinity for bevacizumab (5), and studies on effects of anti-VEGF treatment have been performed using other methods. In newborn mice, partial inactivation of VEGF led to increased mortality, impaired general growth and growth of organs, especially the liver (19) and in 24 days old mice to disturbed cartilage remodelling (20).

Pharmacokinetic studies have shown that rhuMab VEGF (=bevacizumab) was cleared from the serum in a biphasic manner with an initial half-life of 1.2 h in mice and 7 h in rats, and the terminal half-life was 1–2 weeks (5).

Two of the most common adverse effects seen in adults receiving bevacizumab for cancer are proteinuria and hypertension. In mice, local ongoing VEGF production of podocytes in the kidney is necessary for the functioning of the adult glomerular filtration barrier and altered glomerular permeability appeared to be a direct consequence of VEGF inhibition in one study (21).

In rats, serum concentrations of bevacizumab after intravitreal injections were higher in animals with branch retinal vein occlusion than in healthy animals (22), indicating that a breakdown of the blood–retinal barrier allows larger amounts of the drug to enter the general circulation.

### Rabbits

Rabbits have a sparsely vascularized retinas and their VEGF has reduced affinity for bevacizumab, about a fifth of that of primates [Ferrari personal communication in (5)]. Using radiolabelled rhuMab VEGF (=bevacizumab) intravenously in rabbits (5), the distribution indifferent organs could be studied. Radioactivity in plasma was found to be tenfold higher than in tissues where the organs exhibiting the highest radioactivity concentration after 2 h in decreasing rank order were kidney, testes, spleen, heart, lung and thymus with lower levels in brain and eye.

A few pharmacokinetic studies of intravitreal bevacizumab have been performed in rabbits of which one deals with newborn animals (23–25). In rabbit pups, who received 1.25 mg of intravitreal bevacizumab 8 days previously, serum concentrations were significantly higher in 2 weeks old animals (19 300 ng ± 8100 ng/mL) than in 6 weeks old animals (4400 ± 1300 ng/mL; Table 1) (23).

**Table 1:** Bevacizumab concentrations in different compartments after intravitreal injection in one eye

Species	Weight (kg)	Age (weeks)	Dose (mg)	Days after injection	Compartment	Bevacizumab concentration (ng/mL)	References
Rabbit	1.7–2.0		1.25	1	Vitreous	400 000	24
				30	Vitreous	>10 000	
				8	Serum	3300	
Rabbit	1.9–2.5		1.25	14	Plasma	2087	25
Rabbit (pup)	?	2	1.25	8	Serum	19 400 ± 8100	23
		6		8	Serum	4400 ± 1300	
Macaque	3.9–5.5		1.25	7	Serum	1430 ± 186	30
				8 weeks	Serum	67.1 ± 24.3	

In male rabbits (1.7–2.0 kg), intravitreal injection of 1.25 mg bevacizumab in one eye resulted in a peak concentration of 400 000 ng/mL in the vitreous after 1 day, concentration declined with a half-life of 4.32 days and >10 000 ng/mL was maintained for 30 days (Table 1). In serum, a maximum concentration of 3300 ng/mL was found 8 days after injection and it declined with a half-life of 6.86 days. In the vitreous of the fellow eye, bevacizumab concentrations increased from 0.35 ng/mL day 1 to 11.17 ng/mL at 4 weeks (24).

In a study by Nomoto et al., intravitreal injection of 1.25 mg in one eye of rabbits weighing 1.9–2.5 kg each resulted in a maximal concentration in plasma of 2087 ± 2008 ng/mL at 2 weeks with a half-life of 1.85 weeks (Table 1). In the fellow eyes, bevacizumab from the systemic circulation resulted in concentrations in the retina/choroid which were maintained above IC_50_ for 8.0 weeks (25).

In the eye, bevacizumab has been found to be tolerated well (26,27), although a dose-dependent increase in apoptosis has been revealed in photoreceptors and other cells (28,29). Full retinal thickness penetration was found 24 h after injection of 2.5 mg but not after 4 weeks (27).

### Nonhuman primates

Early pharmacokinetic studies of intravenous administration of rhuMab VEGF (=bevacizumab) in cynomolgus monkeys showed that the antibody was cleared from the serum in a biphasic manner with an initial half-life of 11–26 h and a terminal half-life of 1–2 weeks and that the terminal phase was dominant (5).

**Figure 1:**
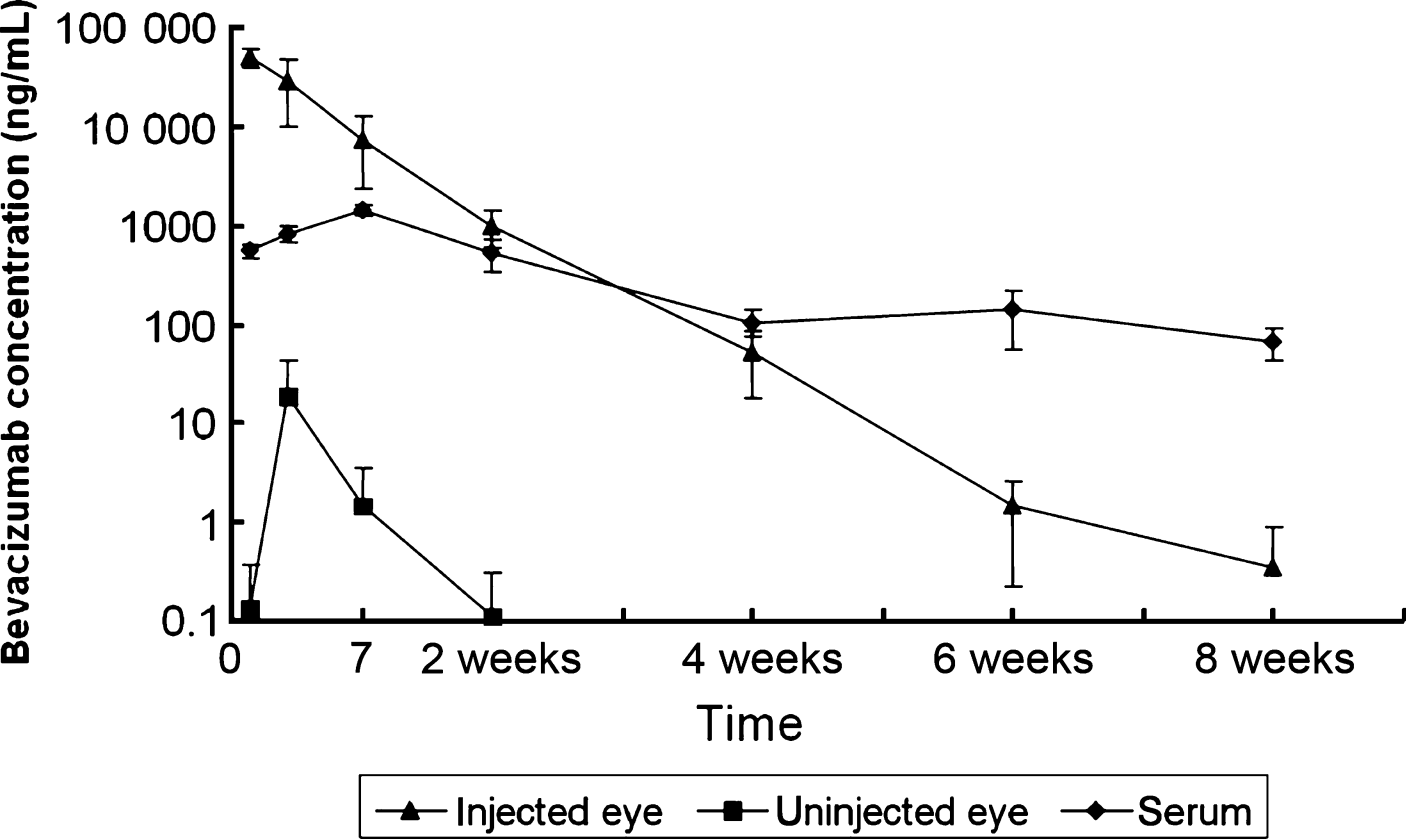
Bevacizumab concentrations in injected eye, uninjected eye and serum after intravitreal injection of 1.25 mg in one eye of adult cynomolgus macaques. From Miyake et al. (30) with the publisher‘s permission.

Intravitreal injection of 1.25 mg bevacizumab in one eye of three male adult cynomolgus macaques (3.9–5.5 kg) resulted in maximum serum concentrations after 1 week of 1430 ± 186 ng/mL (Table 1). Reduction rate was low, and at 8 weeks, serum concentrations were 67.1 ± 24.3 ng/mL, which was approximately 187 times higher than that of the aqueous humour of the treated eye. The effect on serum VEGF concentrations could not be studied because they were below the limit of detection (0.031.2 ng/mL) throughout the experiment (Fig. 1) (30).

In another study on cynomolgus macaques, intravitreal bevacizumab penetrated through the retina and was found in choroidal vessels throughout the experiment indicating substantial transfer of the drug to the blood circulation. Enrichment of bevacizumab was found in rod photoreceptors and endothelial cells of blood vessels (31). In addition, a reversible reduction in the number of choriocapillaris endothelial cell fenestration has been found for at least 2 weeks after intravitreal injection of 1.25 mg bevacizumab as well as choriocapillaris perfusion disturbances (32).

Interestingly, a recent report demonstrates pronounced sustained choroidal vascular involution during the development of ROP (33).

### Humans

In adults with proliferative diabetic retinopathy (PDR), patients treated with intravitreal injection of 1.25 mg bevacizumab in one eye before vitrectomy had significantly lower serum VEGF 7 days after treatment (34). In another study of adults with PDR, different doses (0.0062, 0.0125, 0.062, 0.125, 0.625 and 1.25 mg) of bevacizumab were injected intravitreal, and consistent biologic effects were noted at all doses. A possible therapeutic effect in fellow eyes was found in single patients receiving 1.25 mg in one eye, also indicating that systemic inhibitory concentrations can be achieved in adults (8). We have found no studies of bevacizumab or its effect on VEGF in serum or plasma in preterm infants or in immature animals except the one on rabbit pups by Wu et al. (23).

## Discussion

Bevacizumab enters the general circulation and stays there for weeks to months. It also reaches the fellow eye in potentially therapeutic concentrations (Fig. 1). Young age (23), possibly due to smaller size, and impaired blood–retinal barrier (22) increase serum concentrations. Intravitreal bevacizumab has been reported to reduce serum levels of VEGF in adults (34).

In the cell culture study by Wang et al. (17), 500 ng/mL of bevacizumab was able to neutralize 50 ng/mL of VEGF. Vitreous VEGF levels in type I ROP are unknown, but in vascularly active stage 4 ROP eyes, Sonmez et al. (35) found median (range) concentrations of 3.454 (0.774–8.882) ng/mL which were significantly higher than 0.316 (0.105–0.665) ng/mL in vascularly inactive stage four eyes and 0.059 (0.038–0.135) ng/mL in controls. In stage 5 ROP, levels of 0.119 ± 0.66 ng/mL have been found (36). Bakri et al. (24) found intravitreal bevacizumab concentrations of 400 000 ng/mL after 1 day and >10 000 ng/mL 30 days after injection of 1.25 mg. If similar concentrations are achieved in infant eyes, the doses currently used appear to be very high. Avery et al. (8) found consistent effects on PDR of intravitreal injection of doses as low as 0.006 mg of bevacizumab.

In contrast to the adult healthy macaque, the preterm infant with proliferative ROP has a compromised blood–retinal barrier that may allow more bevacizumab to enter the blood stream. Assuming that a preterm infant with type I ROP at 30–36 weeks is about half the size of an adult macaque of 3.9–5.5 kg and that the serum concentrations reached after an intravitreal injection of 0.625 mg would be similar or, more likely, higher than in the monkey receiving 1.25 mg, serum concentrations after 1 week would be roughly 1400 ng/mL and after 8 weeks 70 ng/mL in the baby (30). Systemic VEGF concentrations in preterm infants show a large variation. Pieh et al. (10) found median (range) plasma concentrations of 0.90 (0.14–2.35) ng/mL and 0.34 (0.07–1.33) ng/mL at 32 and 36 week's PMA, respectively, in infants with ROP. One must, therefore, suspect that serum bevacizumab levels 8 weeks after intravitreal injection still prevents VEGF from acting in preterm infants at a stage when VEGF is needed for the development of kidneys, lungs, brain and other organs.

Very preterm infants at risk for severe ROP have subnormal functioning of many organ systems for the rest of their lives. Anti-VEGF treatment may have the capacity to reduce their reserves even further. These effects may not be obvious until decades after treatment. For clinical off-label use of a drug, basic research and animal experiments are required to evaluate its safety and to reveal potential adverse effects. It is important to note that, in most patients, type I ROP regresses after laser, which has been used for many years with proven efficacy except in the most severe cases. The effects of laser treatment are limited to the eye.

The antibody fragment ranibizumab (Lucentis®, Novartis, Basel, Switzerland), which has a shorter half-life in serum in monkeys 3.5 days (37) than bevacizumab (12.3 ± 2.6 days) (30) and was developed because of concerns of systemic adverse effects of bevacizumab, may be an alternative to study further although 40-fold more expensive.

We suggest that:

1. Experimental studies on animal species with VEGF that binds to bevacizumab (primates, pigs) at the developmental stages corresponding to the human third trimester regarding adverse effects on developing organs such as kidneys, lungs and brain are performed.
2. Infants with very severe ROP who are treated with bevacizumab after laser failure should be included in controlled pharmacokinetic, dose/efficacy and safety trials with close monitoring of serum concentrations of VEGF.
3. Until the above-mentioned studies have been performed, infants who can be treated successfully with laser should not receive anti-VEGF.

Full information about the lack of evidence for safety and efficacy should be given to parents before preterm infants are treated with intravitreal bevacizumab.

## Abbreviations

BPD: Bronchopulmonary dysplasia
HUVEC: Human umbilical vein endothelial cell
IC_50_: Half maximal inhibitory concentration
NO: Nitric oxide
PDR: Proliferative diabetic retinopathy
PMA: Postmenstrual age
rhuMab: Recombinant humanized monoclonal antibody
ROP: Retinopathy of prematurity
VEGF: Vascular endothelial growth factor

## References

[1] Reynolds JD (2011). Bevacizumab for retinopathy of prematurity. N Engl J Med.

[2] Pulido JS (2010). Monitoring systemic complications of intraocular medications. Can J Ophthalmol.

[3] Micieli JA, Micieli A, Smith AF (2010). Identifying systemic safety signals following intravitreal bevacizumab: systematic review of the literature and the Canadian Adverse Drug Reaction Database. Can J Ophthalmol.

[4] Mintz-Hittner HA, Kennedy KA, Chuang AZ (2011). Efficacy of intravitreal bevacizumab for stage 3+ retinopathy of prematurity. N Engl J Med.

[5] Lin YS, Nguyen C, Mendoza JL, Escandon E, Fei D, Meng YG (1999). Preclinical pharmacokinetics, interspecies scaling, and tissue distribution of a humanized monoclonal antibody against vascular endothelial growth factor. J Pharmacol Exp Ther.

[6] Ferrara N (2004). Vascular endothelial growth factor: basic science and clinical progress. Endocr Rev.

[7] Mitchell P (2011). A systematic review of the efficacy and safety outcomesof anti-VEGF agents used for treating neovascular age-related macular degeneration: comparison of ranibizumab and bevacizumab. Curr Med Res Opin.

[8] Avery RL, Pearlman J, Pieramici DJ, Rabena MD, Castellarin AA, Nasir MA (2006). Intravitreal bevacizumab (Avastin) in the treatment of proliferative diabetic retinopathy. Ophthalmology.

[9] Iturralde D, Spaide RF, Meyerle CB, Klancnik JM, Yannuzzi LA, Fisher YL (2006). Intravitreal bevacizumab (Avastin) treatment of macular edema in central retinal vein occlusion: a short-term study. Retina.

[10] Pieh C, Agostini H, Buschbeck C, Krüger M, Schulte-Mönting J, Zirrgiebel U (2008). VEGF-A, VEGFR-1, VEGFR-2 and Tie2 levels in plasma of premature infants: relationship to retinopathy of prematurity. Br J Ophthalmol.

[11] Simon M, Grone HJ, Johren O, Kullmer J, Plate KH, Risau W (1995). Expression of vascular endothelial growth factor and its receptors in human renal ontogenesis and in adult kidney. Am J Physiol.

[12] Eremina V, Sood M, Haigh J, Nagy A, Lajoie G, Ferrara N (2003). Glomerular-specific alterations of VEGF-A expression lead to distinct congenital and acquired renal diseases. J Clin Invest.

[13] Hislop AA, Wigglesworth JS, Desai R (1986). Alveolar development in the human fetus and infant. Early Hum Dev.

[14] Thebaud B (2007). Angiogenesis in lung development, injury and repair: implications for chronic lung disease of prematurity. Neonatology.

[15] Compernolle V, Brusselmans K, Acker T, Hoet P, Tjwa M, Beck H (2002). Loss of HIF-2alpha and inhibition of VEGF impair fetal lung maturation, whereas treatment with VEGF prevents fatal respiratory distress in premature mice. Nat Med.

[16] Sentilhes L, Marret S, Leroux P, Gonzalez BJ, Laquerrière A (2010). Vascular endothelial growth factor and its high-affinity receptor (VEGFR-2) are highly expressed in the human forebrain and cerebellum during development. J Neuropathol Exp Neurol.

[17] Wang Y, Fei D, Vanderlaan M, Song A (2004). Biological activity of bevacizumab, a humanized anti-VEGF antibody in vitro. Angiogenesis.

[18] Klettner A, Roider J (2008). Comparison of bevacizumab, ranibizumab, and pegaptanib in vitro: efficiency and possible additional pathways. Invest Ophthalmol Vis Sci.

[19] Gerber HP, Hillan KJ, Ryan AM, Kowalski J, Keller GA, Rangell L (1999). VEGF is required for growth and survival in neonatal mice. Development.

[20] Gerber HP, Vu TH, Ryan AM, Kowalski J, Werb Z, Ferrara N (1999). VEGF couples hypertrophic cartilage remodeling, ossification and angiogenesis during endochondral bone formation. Nat Med.

[21] Eremina V, Jefferson JA, Kowalewska J, Hochster H, Haas M, Weisstuch J (2008). VEGF inhibition and renal thrombotic microangiopathy. N Engl J Med.

[22] Chuang LH, Wu WC, Yeung L, Wang NK, Hwang YS, Chen KJ (2011). Serum concentration of bevacizumab after intravitreal injection in experimental branch retinal vein occlusion. Ophthalmic Res.

[23] Wu WC, Lai CC, Chen KJ, Chen TL, Wang NK, Hwang YS (2010). Long-term tolerability and serum concentration of bevacizumab (avastin) when injected in newborn rabbit eyes. Invest Ophthalmol Vis Sci.

[24] Bakri SJ, Snyder MR, Reid JM, Pulido JS, Singh RJ (2007). Pharmacokinetics of intravitreal bevacizumab (Avastin). Ophthalmology.

[25] Nomoto H, Shiraga F, Kuno N, Kimura E, Fujii S, Shinomiya K (2009). Pharmacokinetics of bevacizumab after topical, subconjunctival, and intravitreal administration in rabbits. Invest Ophthalmol Vis Sci.

[26] Manzano RP, Peyman GA, Khan P, Kivilcim M (2006). Testing intravitreal toxicity of bevacizumab (Avastin). Retina.

[27] Shahar J, Avery RL, Heilweil G, Barak A, Zemel E, Lewis GP (2006). Electrophysiologic and retinal penetration studies following intravitreal injection of bevacizumab (Avastin). Retina.

[28] Inan UU, Avci B, Kusbeci T, Kaderli B, Avci R, Temel SG (2007). Preclinical safety evaluation of intravitreal injection of full-length humanized vascular endothelial growth factor antibody in rabbit eyes. Invest Ophthalmol Vis Sci.

[29] Avci B, Avci R, Inan UU, Kaderli B (2009). Comparative evaluation of apoptotic activity in photoreceptor cells after intravitreal injection of bevacizumab and pegaptanib sodium in rabbits. Invest Ophthalmol Vis Sci.

[30] Miyake T, Sawada O, Kakinoki M, Sawada T, Kawamura H, Ogasawara K (2010). Pharmacokinetics of bevacizumab and its effect on vascular endothelial growth factor after intravitreal injection of bevacizumab in macaque eyes. Invest Ophthalmol Vis Sci.

[31] Heiduschka P, Fietz H, Hofmeister S, Schultheiss S, Mack AF, Peters S (2007). Penetration of bevacizumab through the retina after intravitreal injection in the monkey. Invest Ophthalmol Vis Sci.

[32] Peters S, Heiduschka P, Julien S, Ziemssen F, Fietz H, Bartz-Schmidt KU (2007). Ultrastructural findings in the primate eye after intravitreal injection of bevacizumab. Am J Ophthalmol.

[33] Shao Z, Dorfman AL, Seshadri S, Djavari M, Kermorvant-Duchemin E, Sennlaub F (2011). Choroidal involution is a key component of oxygen induced retinopathy. Invest Ophthalmol Vis Sci.

[34] Qian J, Lu Q, Tao Y, Jiang YR (2011). Vitreous and plasma concentrations of apelin and vascular endothelial growth factor after intravitreal bevacizumab in eyes with proliferative diabetic retinopathy. Retina.

[35] Sonmez K, Drenser KA, Capone A, Trese MT (2008). Vitreous levels of stromal cell-derived factor 1 and vascular endothelial growth factor in patients with retinopathy of prematurity. Ophthalmology.

[36] Velez-Montoya R, Clapp C, Rivera JC, Garcia-Aguirre G, Morales-Cantón V, Fromow-Guerra J (2010). Intraocular and systemic levels of vascular endothelial growth factor in advanced cases of retinopathy of prematurity. Clin Ophthalmol.

[37] Gaudreault J, Fei D, Rusit J, Suboc P, Shiu V (2005). Preclinical pharmacokinetics of ranibizumab (rhuFabV2) after a single intravitreal administration. Invest Opthalmol Vis Sci.

